# Exploring the Immediate Effects of Nadi Shuddhi Pranayama on Heart Rate Variability Among Young Adults

**DOI:** 10.1007/s10484-025-09710-4

**Published:** 2025-04-24

**Authors:** Hirok Chakraborty, A. V. Vinay, R. Sindhu, Ratnesh Sinha

**Affiliations:** 1 Department of Physiology, Manipal Tata Medical College, Manipal Academy of Higher Education, Manipal, India; 2 Department of Community Medicine, Manipal Tata Medical College, Manipal Academy of Higher Education, Manipal, India

**Keywords:** Yoga, Nadi Shuddhi Pranayama, Heart rate variability, Blood pressure, Autonomic function

## Abstract

The young working population experiences a high level of perceived stress. Stress distorts our sympathovagal balance which maintains homeostasis in our body and leads to stress related diseases. This stress can be reduced by practicing yoga, which has various components that can bring a sensation of calmness and increased awareness. Heart rate variability (HRV) being a non-invasive technique is a good method for assessing sympathovagal balance. The present study aimed to determine the immediate changes in HRV in young adults following Nadi Shuddhi Pranayama (NSP). The study was initiated in the Physiology Department of our medical college. 40 naive to pranayama, volunteers of both genders aged 20–40 years were recruited for the study. Their perceived stress scale (PSS) score and ECG in lead II were recorded. Before ECG recording and NSP performance, volunteers were demonstrated the NSP technique by a certified yoga trainer. A 15-minute baseline ECG recording pre-NSP, followed by a 15-minute ECG recording post-NSP for HRV analysis in recovery phase was attained using a digital polygraph. Blood pressure was noted before and at the end of ECG recording. Analysis of variability in HRV was done before and after the performance of NSP. Compared to baseline readings, a significant decrease in systolic blood pressure (SBP) and a significant increase in HRV parameters like SDNN (standard deviation of RR Intervals adjacent normal to normal intervals), and HF (high frequency), along with a significant decrease LF (low frequency), LF/HF (low/high frequency) ratio was observed post NSP practice. *p* < 0.05 was considered as significant. We conclude that the practice of NSP even for short period can shift the balance of the ANS toward the PNS.

## Introduction

The current world scenario keeps almost every person under some degree of stress, which has directly or indirectly impacted his/her physical, mental and social wellbeing and has resulted in increased morbidity and mortality (Crosswell et al., 2020).

Stress has been characterized as the body’s response to disruption caused by a stimulus, that aims to upset the body’s equilibrium and stability, both mentally and physically (Selye, 1956). Stress has also been described as an unhealthy condition in which the sympathetic nervous system (SNS) is disproportionately stimulated, causing acute or chronic physical, psychological, and behavioural impairment (Kim et al., 2018). Low-level stress may offer some health benefits, but excessive stress can cause strokes, heart attacks, and psychological impairment (Herbert, 1997).

The American Psychological Association defined stress as “the pattern of specific and nonspecific responses an organism makes to stimulus events that disturb its equilibrium and tax or exceed its ability to cope” (Castaldo et al., 2019).

Stress influences our ability to judge and make rational decisions and has been shown to reduce our performance (Castaldo et al., 2017). Workplace stress can impede our efficiency and potentially have catastrophic repercussions on others, exemplified by a pilot’s excessive flight hours, where the stress may jeopardise the safety of passengers if he fails to perform his flying duties adequately (Castaldo et al., 2015). Mental stress modifies our ability to react to external stimuli by affecting the activities of the autonomic nervous system (ANS) which is responsible for all our reactions to any stimulus (Castaldo et al., 2015). As mental stress is perceived by an individual internally which causes mental strain and fatigue; so, it is not easy to measure it. (Herbert, 1997). Till date, there are no known validated parameters that can be used to measure the mental stress levels of individuals. In the past few years though, Heart rate variability (HRV) has started gaining popularity in being a good indicator for assessing mental stress levels in individuals (Castaldo et al., 2017). HRV signifies the variations of both instantaneous heart rate and the series of inter-times between consecutive peaks of the R-wave (RR intervals) of the Electrocardiogram (ECG) (T. Force 1996). The cardiac-vagal spectral component of HRV may be sensitive to the recent experience of persistent emotional stress, regardless of their level of physical fitness (Dishman et al., 2000). The significance of HRV concerning stress is so great that Park et al., (2019) recommended that assessing stress in early adulthood would yield more accurate results if HRV analysis is conducted simultaneously.

The SNS enhances the rate and force of the heart during exercise and in response to mental or physical stress, which is associated with a reduction in HRV. In contrast, the parasympathetic nervous system (PNS) slows the heart rate and increases HRV to restore homeostasis. This dynamic interplay between the SNS and PNS enables the heart to rapidly adjust to varying circumstances and demands needs based on the context (Thayer et al., 2012). Thus, higher HRV associated with greater parasympathetic activity, is suggestive of better self-regulation and is largely affiliated with lower cardiovascular risk. (Brinkmann et al., 2020).

Regular practice of yoga has found to be beneficial in stabilizing autonomic nervous system and achieve metal calmness and stability. (Ghiya, S et al., 2012) The practice of yoga commonly includes set of asanas, pranayama and meditation. Slow breathing exercises, such as Pranayama methods, significantly influence heart rate variability (HRV), indicating the equilibrium between the sympathetic and parasympathetic divisions of the autonomic nervous system. Elevated HRV correlates with improved cardiovascular health, emotional modulation, and stress resilience (Lehrer & Gevirtz, 2014). Among different pranayama techniques available, Nadi Shuddhi Pranayama (NSP), or “alternate nostril breathing,” is a yogic method aimed at harmonising the body’s vital forces through the purification of the nadis (subtle energy pathways). Originating from ancient yogic traditions, it entails deliberate, alternating intake and exhalation via the nostrils (Saraswati, 1984). Practice of yoga synchronises the sympathetic and parasympathetic nerve systems, fostering bodily and mental balance (Streeter et al., 2012).

Nadi Shuddhi is frequently employed in the preliminary phases of yoga, facilitating mental tranquilly, improving concentration, and priming practitioners for profound contemplative experiences. Its importance is in its capacity to alleviate stress, regulate autonomic processes, and promote overall well-being (Nivethitha et al., 2017) Slow breathing practices like NSP stabilize our autonomic control system and have been advocated for relaxing and relieving stress (Dhanvijay et al., 2018). The benefits of practicing NSP for improving well-being, memory recall, stress relief, and physical relaxation was demonstrated by Joshi et al. (2011).

Given the significant amount of stress experienced by the young working population (Law et al., 2020) We intended to explore and identify a simple non-invasive, non-pharmacological bio-feedback techniques that would help in reducing the stress immediately and help to achieve autonomic balance. NSP is one such technique that we came across that can be utilized for the benefit of immediate reduction of stress among individuals. NSP is usually practiced as a part of integrated yoga module. To quantify the exclusive effects NSP, this study was designed. Here we could document the quanta of change a 15-minutes practice of NSP can induce in the HRV parameters, sheading insight of its impact on autonomic function.

## Methodology

The interventional study (registered with CTRI - CTRI/2023/05/052459) was conducted in the Department of Physiology of a medical college for 8 months after getting permission from the ethical committee of the host institution. Volunteers were recruited from the working population associated with the medical college.

### Sample Size: 40

The required sample size of 40 was calculated using Cohen’s formula for the effect size of 0.46 based on the study by Pal et al. (2004). between pairs, a power of 80%, and a level of significance of 5% (two sided) using an online Stimulator sample size calculator (Dhand et al., 2014).

### Selection Criteria for the Study Volunteers

#### Inclusion Criteria

All clinically healthy volunteers in the age group of 20–40 years, who showed interest to take part in the study (random sampling technique), had a PSS score > 13, and were naive to the practice of pranayama were selected for the study.

#### Exclusion Criteria

Anyone who is a yoga or meditation practitioner, suffering from chronic diseases like hypertension, diabetes, diseases of the thyroid gland, or having mechanical or infective nasal blockage was exempted from participating in the study. Further some, chronic smokers, alcoholics, drug addicts, and individuals on antipsychotic drugs were excluded from the study.

### Tools Used

- Reliable and validated Perceived stress scale (PSS) form (Baik et al., 2019).
- Anthropometric and demographic parameters were noted along with a self-administered questionnaire that recorded the data on addiction, allergy, chronic diseases, etc.
- Mercury-free sphygmomanometer model no: BPDG041 by Diamond recorded blood pressure.
- Wall-mounted measuring tape for height in centimetres (cm).
- Digital weighing scale in kilograms (kg) measured weight.
- Body mass index (BMI) was derived from height and weight (Frankel HM et al., 1992),
- RMS digital polygraph recorded and analysed lead II electrocardiogram (ECG) for HRV parameters. The time domain parameters include standard deviation of RR Intervals adjacent normal to normal intervals (SDNN), root mean square of differences between successive RR intervals (RMSSD). The frequency domain parameters, were derived through Fast Fourier transformation of the ECG data to express the values in total power of Low frequency (LF) in ms^2^, High frequency (HF) in ms^2^ and LF/HF ratio. The LF and HF can also be expressed in normalized unit (nu). and mean heart rate (HR).

###  Description of the Procedure

Following the permission granted by the institutional ethical committee, individuals who showed interest and consented (in writing) to take part in the study were asked to report to the Department of Physiology at 08:30 am after a sound sleep of a minimum of 6 h on the previous night and abstaining from intense physical activity. Additionally, volunteers were told to have a light breakfast free of caffeine, 1.5–2 h prior to arrival in the department where they were required to perform NSP. Upon arrival in the department, volunteers were requested to complete the PSS questionnaire. Volunteers with PSS scores > 13 indicating moderate stress were selected to take part in the study.

Perceived stress scale (PSS) questionnaire: The PSS is a 10-item self-report questionnaire that assesses how a person perceived stressfulness situations in the past 1 month of their lives. Questions are based on a 5-point Likert scale. For each question, the participant had to choose and tick the appropriate option from the following alternatives: 0 - never, 1 - almost never, 2 - sometimes, 3 - fairly often, and 4 - very often. In the present study, PSS was the inclusion criteria hence all the volunteers completed the questionnaire. Individual scores on the PSS range from 0 to 40 with, value 0–13: Low Stress, 14–26: Moderate Stress, 27–40: high stress (Cohen, S et al., 1983).

The volunteers with PSS score > 13 were included in the study, volunteers filled out a self-administered questionnaire to document data on acute and chronic diseases, addiction, allergies and other relevant factors. This information further sorted the inclusion and exclusion criteria for the study. Subsequently, anthropometric and demographic parameters were noted. Standing height was measured using a wall-mounted measuring tape, while weight was recorded using a digital weighing scale.

With the volunteers sitting comfortably, arterial blood pressure was measured using a Mercury-free manual sphygmomanometer (model no: BPDG041 by Diamond). After that, the ECG leads were attached for Lead II ECG recording using a polygraph machine (RMS, India). Before initiating ECG recordings, a certified yoga trainer demonstrated the NSP technique, which involved inhaling and exhaling through one nostril at a time, as outlined below:

The process of NSP began with exhalation through the left nostril while occluding the right nostril with the right thumb, followed by inhalation through the left nostril. This was succeeded by expiration through the right nostril, keeping the left nostril sealed by the right ring finger. After exhaling air through the right nostril, inhalation occurred through the same (right) nostril, and this was followed by exhalation through the left nostril, again occluding the right nostril (Bhavanani et al., 2014).

**Fig. 1:**
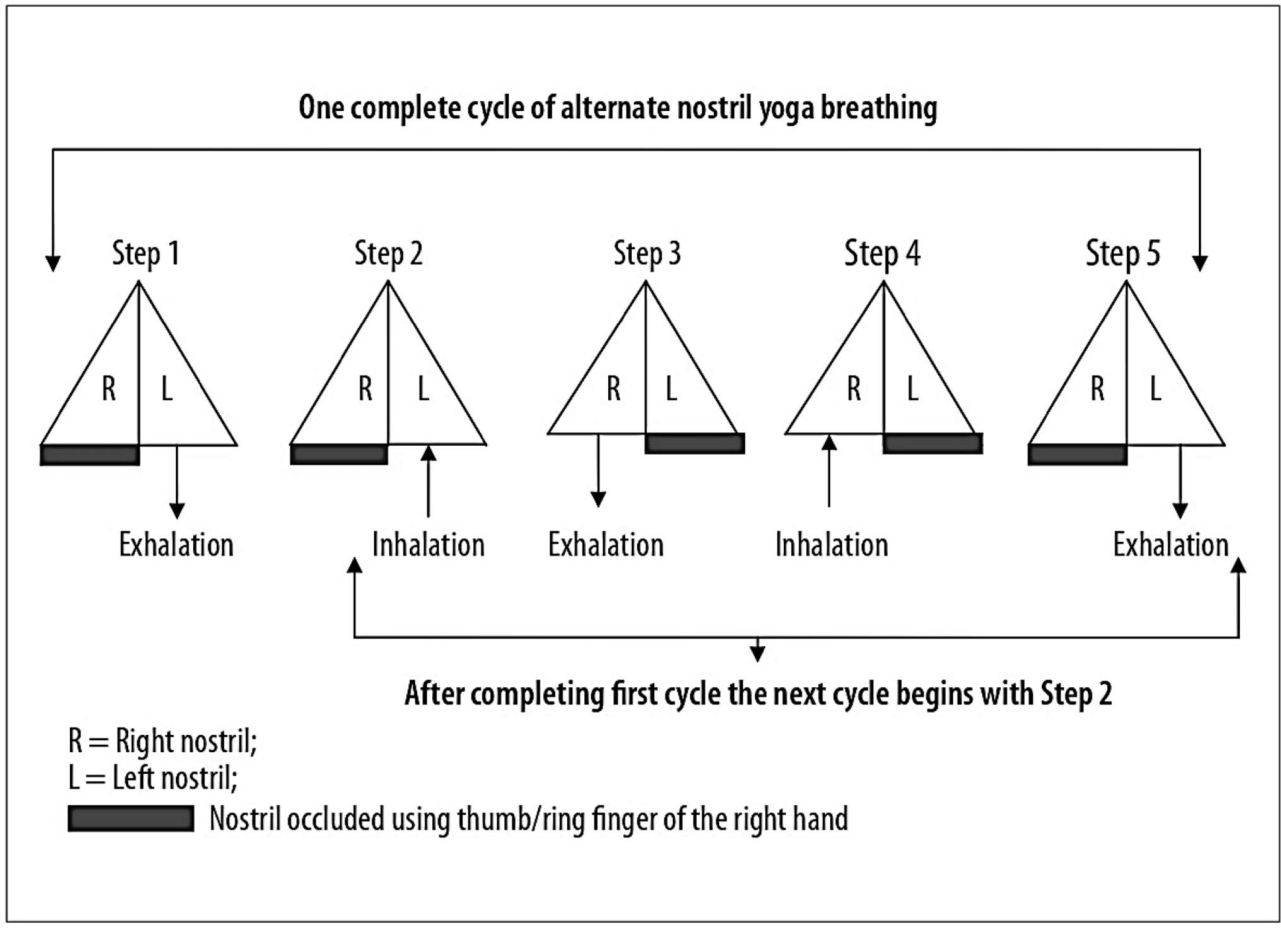
Process of NSP

This entire sequence which started and ended with the exhalation by the left nostril (including exhalation and inhalation by the right nostril) constituted 1 cycle of NSP (Telles et al., 2014) as depicted in Fig. 1. The volunteers completed one cycle of NSP in 15 s i.e., 4 cycles per minute. Volunteers performed 2–3 trial breathings after the demonstration of the pranayama.

The Volunteer was made to sit comfortably on a couch and rest for 10 min, The ECG lead would be connected, and the ECG waves were monitored to rule out any abnormalities. Once it’s confirmed that ECG was normal, A 15-minute baseline recording of the ECG in lead II was then obtained using RMS digital polygraph. An additional 15-minute ECG recording was done in the recovery phase following the 15-minute NSP performance. Blood pressure was recorded just before the ECG recording and again at the end of relaxation i.e.15 min after NSP.

#### ECG & HRV Data Acquisition

HRV data was extracted from the ECG recordings in lead II using RMS Vagus HRV software (RMS, India) adhering to the task force guidelines. (T. Force 1996). RMS software modified the ECG after subjecting the parameters to Fast Fourier Transformation (Orsila et al., 2008). The HRV data was extrapolated from the complete 15 min ECG recorded during baseline and recovery phase separately. The ECG recordings were scanned manually for any abnormalities, and only those data without any abnormalities were used for the HRV analysis All the recordings were done on a single visit to the department on the same day.

#### Data Analysis

All the data was compiled in MS Excel sheet and were analysed using Jamovi software version 2.3.31. All the data followed normal distribution and was confirmed by Shapiro-Wilk test and hence the descriptive statistics of frequency, mean, standard deviations were used to represent the data of the study participants. Continuous variables were compared using paired t-test. Statistical significance was set at the *p* < 0.05 level for all analyses.

## Results

A total of 40 volunteers were recruited in our study of which 19 were females and 21 were males. Table 1 demonstrates the mean and standard deviation of the PSS score along with the sociodemographic parameters of the 40 volunteers (both male and female) i.e., age, height, weight and BMI. Since the study was done in one setting and the same volunteers served as controls for the cases, there was no alteration in these parameters.

Table 2 illustrates the results of paired t-test examining the effects of NSP on the Systolic Blood Pressure (SBP), Diastolic Blood Pressure (DBP) and mean heart rate (HR) of the volunteers pre and post NSP. The results revealed a statistically significant decline in the SBP and the mean HR post NSP. DBP post NSP did not show any significant variation.

**Fig. 2:**
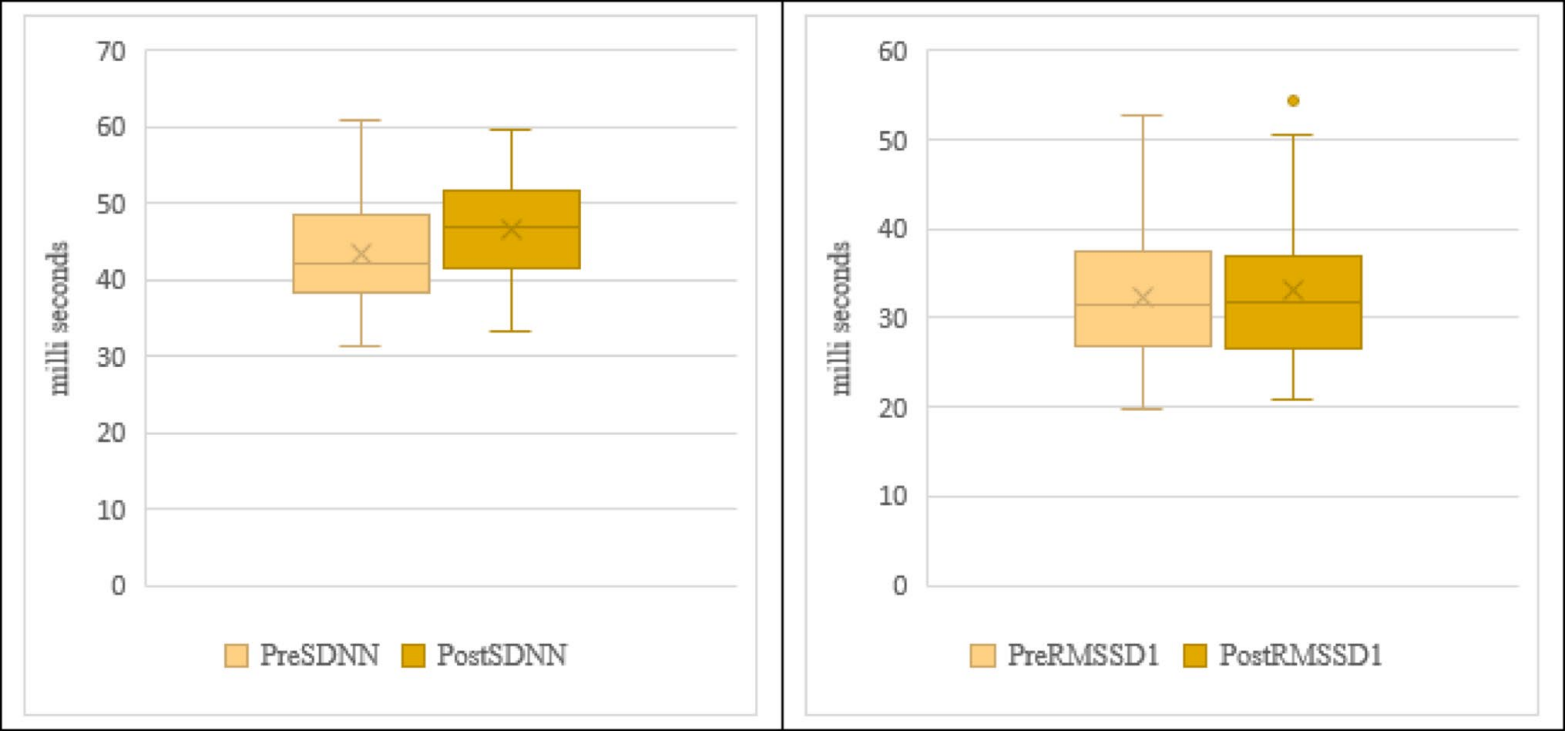
Comparison of Time domain HRV parameters pre and post NSP performance

In Fig. 2 comparisons of time domain HRV parameters are presented in mean and standard deviation. The results indicate a statistically significant increase in the SDNN post-NSP (43.44 ± 7.14 ms to 46.61 ± 6.77 ms), whereas the RMSSD did not show any statistically significant variation (32.22 ± 7.23 to 33.09 ± 7.67).

**Fig. 3:**
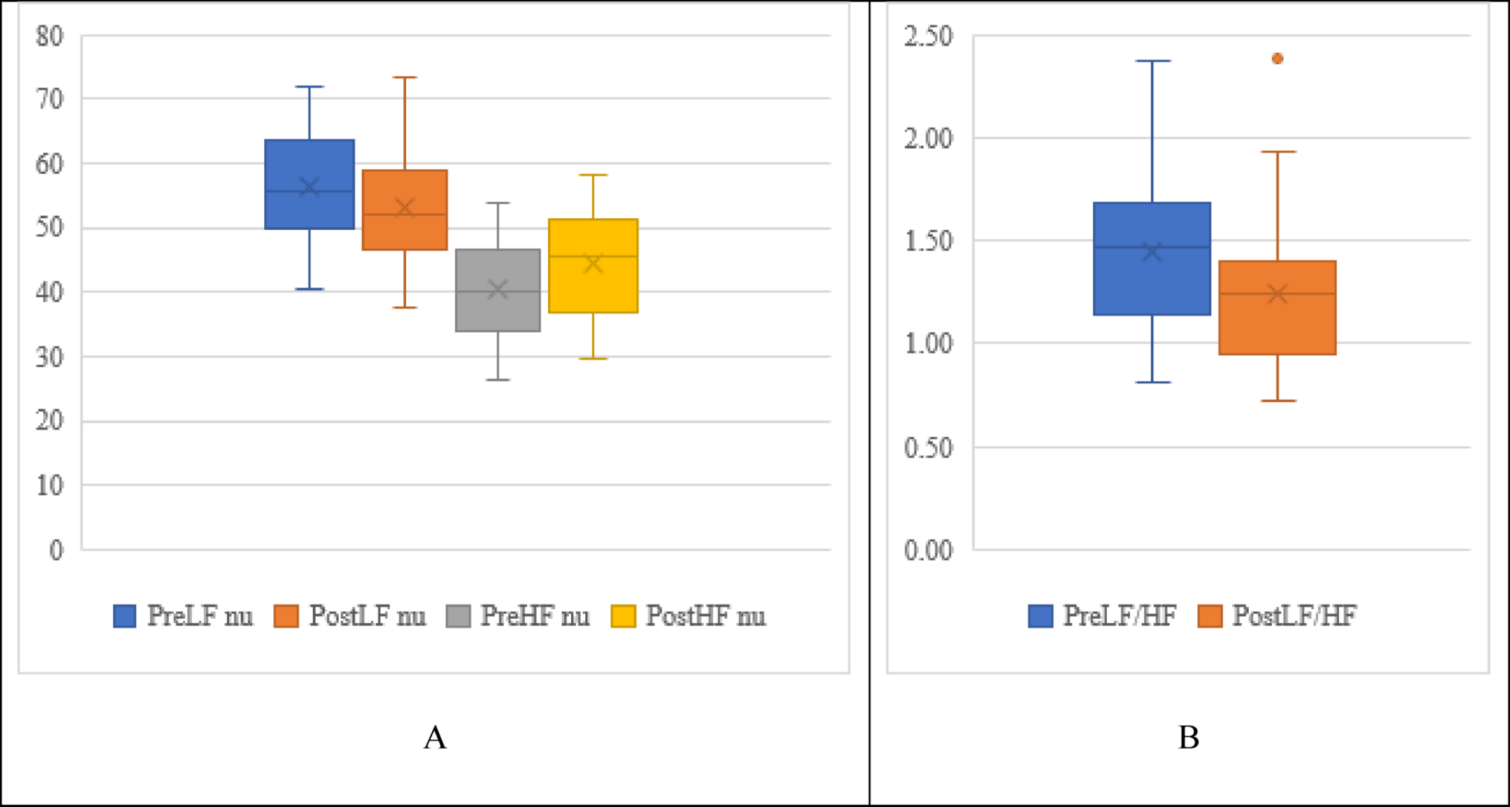
Comparison of Frequency domain HRV parameters pre and post NSP performance

Figure 3 expresses the comparison of frequency domain HRV parameters. The Fig. 3A demonstrates a statistically significant decline in the LF (56.33 ± 8.67 to 53.02 ± 8.78) and a statistically significant increase in the HF (40.54 ± 7.48 to 44.48 ± 7.81) following the NSP performance compared to baseline. Additionally (Fig. 3B), a statistically significant decline in the LF/HF ratio (1.45 ± 0.39 to 1.24 ± 0.35) post NSP can also be observed.

## Discussion

This quantitative study provides in-depth insights into the impact of short-term behavioural intervention in the form of NSP on the autonomic control system of the heart. The findings of this study can guide developing and delivering tailored behaviour change interventions in moderately anxious young adults, which could be extrapolated to clinical practice. The key findings of our study affecting the cardiovascular system (CVS) and HRV parameters following the performance of NSP have been detailed below.

### Cardiovascular Parameters

The results of our study concerning cardiovascular parameters revealed a significant decrease in SBP following the performance of NSP. These findings are consistent with the observations of previous studies (Goel et al., 2016; Saisupriya et al., 2020) where a significant reduction in SBP immediately after the performance of NSP was also documented. Slow breathing increases the vagal modulation of sinoatrial and atrioventricular nodes and enhances baroreceptor sensitivity, which can be attributed to the decline in blood pressure (BP) indices (Vempati et al., 2002), especially the SBP the variation of which is far greater than the DBP in the short term (Goel et al., 2016). Breathing exercise modulates the hypothalamic activity, resulting in a decrease in SNS activity. A higher vagal tone lessens the strain on the heart which subsequently reduces cardiac output (CO) and consequently SBP (Pal et al., 2014). Additionally, the observed decrease in SBP in a previous study (Bodhe et al., 2015) has been attributed to a reduction in sympathetic tone, an increase in parasympathetic tone and a decrease in stress levels. An earlier study (Telles et al., 2017) demonstrated a significant decline in SBP of 4.5 mg following 18- minute performance of NSP, which aligns with our findings of a fall in SBP after 15 min of NSP intervention in healthy adults.

DBP associated with changes in peripheral vascular resistance is mainly determined by SNS. A nonsignificant decline in the DBP evident in our study synchronizes with the findings of Dandekar (2013) who reported similar findings in his research, indicating, that there is no significant change in the peripheral resistance of the tissues during or after the performance of NSP in the short term.

However, some investigators (Turankar et al., 2013) reported no changes in the SBP and DBP following the breathing exercises, which may be attributed to the short duration of their study.

Our study’s significant decrease in mean HR suggests that breathing exercises may stimulate the vasomotor center, leading to increased PNS activity and reduced SNS activity. This shift may contribute to the observed decrease in mean pulse rate (Biswas et al., 2014 and Pal et al., 2014). HR is regulated by both sympathetic and vagal innervation, along with various humoral factors (Telles et al., 2011). Slow breathing is known to increase the vagal modulation of sinoatrial and atrioventricular nodes and enhance baroreceptor sensitivity which might have caused the reduction in HR (Vempati et al., 2002).

The consistency of these findings is supported by previous studies, like that of Jahan et al., (2021) who also reported a significant drop in SBP and HR among medical students aged 18–20 years post yoga practice (alternate nasal breathing exercise).

The CVS parameters of our study indicate the inclination of the ANS towards the PNS, synchronizing with the hypothesis which states that yoga exercises like NSP induce this shift potentially via direct vagal stimulation. This mechanism may facilitate voluntary control over the ANS and contribute to stress relief (Divya TS et al., 2017).

### Time Domain Parameters

A decrease in the power of HRV is indicated by SDNN, which reflects inflexible changes in heartbeats suggestive of sympathetic overactivity. A 24 h SDNN recording is considered the “gold standard” for medical stratification of cardiac risk (T. Force 1996). In short term, SDNN values of HRV are a reliable predictor for morbidity and mortality (Kleiger et al., 1987), and higher values are associated with a greater sense of well-being (Shaffer et al., 2014). Our study found a significant increase in SDNN following the performance of NSP, indicating a predominance of the PNS. This shows that even short-term practice of NSP can shift the ANS balance towards the PNS which helps to cope with day-to-day stresses in life more efficiently. The evidence given by Dhanvijay et al., (2018) vindicates our inference that NSP helps in reducing stress by decreasing the cardiovascular autonomic response in the form of downregulation of SNS.

The insignificant variation in RMSSD observed in our study is echoed by the findings of Bhimani et al. (2011), which suggests that the influence of NSP on HRV may become apparent only over a longer duration and may not have a notable impact on HRV in the short term.

### Frequency Domain Parameters

The LF band of the HRV is mainly related to sympathetic modulation when expressed in normalized units (T. Force 1996) while the efferent vagal activity is a major contributor to the HF band. The LF/HF ratio is correlated with the sympathovagal balance. Thus, the tension of the sympathetic nervous system is associated with the increase in LF & LF/HF ratio.

This study revealed a significant reduction in LF and LF/HF ratio alongside a significant increase in HF of HRV. The results are consistent with an earlier study by Bhimani et al. (2011) which examined 59 first-year healthy MBBS students. The rise in HF, which primarily reflects cardiovagal modulation and is likely due to impulse spillover from the respiratory center, indicating a predominance of parasympathetic effect on the heart. LF is affected by both sympathetic and parasympathetic activity and increases mainly due to sympathetic stimulation. The decrease in LF and an increase in LF/HF indicate a shift in balance towards the vagal activity. (Udupa et al., 2022).

On the contrary, (Raghuraj et al., 2008) observed a significant increase in LF & LF/HF ratio, indicating that HRV changes are not purely related to autonomic balance shift but are apparently due to a link with respiration.

But the article by (Billman, 2013) completely negates the interpretational value of LF/HF stating that LF/HF as a parameter cannot accurately quantify cardiac “sympatho-vagal balance” either in health or disease. Ruling out the importance of the change in LF/HF.

Elevated SDNN and HF are indicative of enhanced parasympathetic function and overall wellbeing. On the contrary, decreased values of SDNN and HF are associated with a greater risk of morbidity and mortality (Tung et al., 2019) and the results of this study signal that short term practice of NSP induces parasympathetic tilting of the ANS.

## Conclusion

We conclude that 15-minute practice of NSP can shift the balance of the ANS towards the parasympathetic predominance. The observed increases in SDNN indicates the overall variability of the heart and increase in HF indicate enhanced parasympathetic outflow. whereas reduction in LF suggests decreased sympathetic drive. Therefore, slow breathing exercises like NSP can be used as a biofeedback tool, in managing stress.

## Limitation

This was a single-time procedure, follow-up with greater number of participants could give better knowledge about the influence of the intervention in short term. The study design did not have the control group, so we could not compare the effect of NSP on HRV in comparison to quiet breathing.

**Table 1:** Mean PSS score, age, height, weight and BMI of the volunteers (N = 40)

Parameters	Mean ± SD
PSS score	20.23 ± 4.12
Age (years)	29.95 ± 5.79
Height (meter)	1.61 ± 0.08
Weight (kg)	63.23 ± 9.17
BMI (kg/meter^2^)	24.54 ± 4.26

**Table 2:** Cardiovascular parameters of the volunteers (N = 40).

Parameter	Pre NSP Mean ± SD	Post NSP Mean ± SD	p-value
Systolic Blood Pressure (mmHg)	113.±11.16	111.2 ± 11.25	0.012*
Diastolic blood pressure (mmHg)	75.2 ± 7.1	74.85 ± 7.54	0.573
Mean HR (beats per minute)	77.80 ± 9.97	76.03 ± 9.39	0.027*

Paired ‘t’ test; p value <0.05 was considered as significant

## Data Availability

The data that support the findings of this study will be made available from the corresponding author upon reasonable request.
